# Relationship Between Postoperative Feeding Status and Salivary Bacterial Load Following Oral Surgical Procedures

**DOI:** 10.7759/cureus.88862

**Published:** 2025-07-27

**Authors:** Teruyuki Niimi, Sakiko Soutome, Akira Imakiire, Hideto Imura, Masahito Hara, Kei-ichiro Miura, Mitsunobu Otsuru, Tomohiro Yamada, Masahiro Umeda, Nagato Natsume

**Affiliations:** 1 Division of Research and Treatment for Oral and Maxillofacial Congenital Anomalies, School of Dentistry, Aichi Gakuin University, Nagoya, JPN; 2 Department of Oral Health, Nagasaki University Graduate School of Biomedical Sciences, Nagasaki, JPN; 3 Department of Oral and Maxillofacial Surgery, Nagasaki University Graduate School of Biomedical Sciences, Nagasaki, JPN; 4 Department of Oral and Maxillofacial Surgery, Nagasaki University, Nagasaki, JPN; 5 Department of Oral and Maxillofacial Surgery, Faculty of Medicine, Academic Assembly, University of Toyama, Toyama, JPN; 6 Department of Oral and Maxillofacial Surgery, Kanagawa Dental University, Yokosuka, JPN

**Keywords:** feeding status, oral surgical procedures, salivary bacterial count, surgical site infections, systemic antibiotic administration

## Abstract

Introduction: Oral and maxillofacial surgery involves clean-contaminated wounds exposed to saliva, increasing the risk of surgical site infections (SSIs). While systemic antibiotics are commonly used postoperatively, the effect of feeding status on salivary bacterial counts remains unclear.

Methods: This prospective observational study included 24 patients (greater than or equal to six years) undergoing oral surgery with postoperative enteral feeding. Salivary bacterial counts were measured preoperatively, during enteral feeding, liquid diet, and regular diet phases using real-time polymerase chain reaction targeting the 16S ribosomal ribonucleic acid gene. Statistical analysis was performed using paired t-tests.

Results: During enteral feeding, all patients received systemic antibiotics, and salivary bacterial counts remained comparable to preoperative levels (101%). In the liquid diet phase, bacterial counts decreased with ongoing antibiotics (88%) but increased without antibiotics (122%). During the regular diet phase, counts returned to baseline (98%).

Conclusion: Systemic antibiotics suppress salivary bacterial growth, while reduced oral intake may increase bacterial burden. Additional oral hygiene measures may be necessary during the transition period to prevent SSIs.

## Introduction

In oral and maxillofacial surgery, surgical wounds are often located within the oral cavity. In general, surgical wounds are classified into four categories based on their level of contamination: Class I (clean), Class II (clean-contaminated), Class III (contaminated), and Class IV (dirty) [1]. Due to the presence of numerous resident bacteria in the oral cavity and the constant contact of saliva with surgical wounds, intraoral wounds are typically classified as Class II: clean-contaminated. Consequently, the incidence of surgical site infections (SSIs) tends to be relatively high [2].

To prevent SSIs following oral and maxillofacial surgery, systemic antibiotics are often administered from the time of surgery through 24-48 hours postoperatively [3,4]. In addition, reducing the bacterial load in saliva is considered important to prevent infections, and maintaining good oral hygiene from the preoperative period is desirable. The main oral resident bacteria responsible for SSIs include Streptococcus species, such as *Streptococcus mitis*, *Streptococcus salivarius*, and *Streptococcus anginosus*, which form biofilms and easily colonize wounds, thereby causing inflammation. Anaerobic gram-negative rods, such as Prevotella, Porphyromonas, and Fusobacterium, are also involved, known for worsening infections and delaying healing [5]. Additionally, Actinomyces and Staphylococcus species, including *Staphylococcus aureus* (with methicillin-resistant *S. aureus* risk), can be present in the oral cavity and contribute to infection [6].

To protect the surgical wound, enteral feeding is sometimes employed postoperatively. However, enteral feeding may reduce the natural self-cleaning mechanisms of the oral cavity, namely salivary secretion, mastication, and swallowing, potentially leading to an increase in bacterial count in saliva. Despite this concern, no previous studies have investigated the changes in salivary bacterial count following oral and maxillofacial surgery.

The primary objective of this preliminary study is to evaluate changes in salivary bacterial counts in relation to different postoperative feeding phases: enteral nutrition, liquid diet, and regular diet, in patients undergoing oral surgery. We also aim to explore how the concurrent use or discontinuation of systemic antibiotics during these phases may influence the bacterial load. This study hypothesizes that salivary bacterial counts would increase during the periods of limited oral intake (e.g., enteral feeding), due to reduced oral self-cleansing, and decrease after the resumption of a regular diet.

## Materials and methods

Study design and participants

This study is a prospective observational study that does not involve any interventions or invasive procedures. The patient enrollment period was from July 8, 2024, to April 30, 2025. The study was conducted at the Department of Oral and Maxillofacial Surgery at Nagasaki University Hospital, Aichi Gakuin University Dental Hospital, and Kanagawa Dental University Hospital.

The inclusion criteria included patients undergoing oral and maxillofacial surgery who would receive enteral nutrition postoperatively, aged six years or older, and patients who provided written informed consent, either directly or through a legally authorized representative. The exclusion criteria included patients who would require postoperative management with tracheostomy or endotracheal intubation, patients with severe cognitive impairment, patients with active oral infections and receiving systemic antibiotics prior to surgery, and patients with severe xerostomia.

Since no similar studies had been conducted previously, no foundational data were available for formal sample size calculation. Accordingly, this investigation was conducted as a preliminary study. A target sample size of 40 patients was determined pragmatically, based on the estimated number of eligible patients during the enrollment period and logistical feasibility, rather than statistical considerations.

Factors examined

The following variables were recorded: age, sex, underlying disease, surgical procedure, operation time, type and duration of prophylactic antibiotic use, and salivary bacterial counts at the preoperative period, enteral feeding period, liquid diet period, and regular diet period.

The salivary bacterial load was quantified using real-time polymerase chain reaction (PCR), following a previously reported method [7]. Briefly, 0.1-0.2 mL of saliva was collected, and genomic DNA was extracted using the InstaGene Matrix (Bio-Rad, Hercules, CA) according to the manufacturer’s instructions. After incubation and heat treatment, the samples were stored at -20°C until analysis. For quantification, samples were thawed and centrifuged, and the supernatant was subjected to quantitative real-time PCR using KOD SYBR® qPCR Mix (TOYOBO Co., Ltd., Osaka, Japan). Primers targeting the 16S ribosomal ribonucleic acid gene were used, and bacterial copy numbers were estimated based on standard calibration curves generated using synthesized target DNA sequences. PCR conditions and data analysis were performed as previously described [7].

Statistical analysis

The logarithmic values of bacterial counts followed a parametric distribution, as confirmed by the Shapiro-Wilk test. Paired t-tests were used to compare bacterial counts between the preoperative period and each of the following stages: enteral nutrition, liquid diet, and solid diet (three pairwise comparisons). Due to the preliminary and exploratory nature of the study, no correction for multiple testing was applied to reduce the risk of Type II error. For each comparison, effect sizes (Cohen’s d) and 95% confidence intervals were calculated to evaluate the magnitude and precision of the observed differences. A two-tailed p-value of <0.05 was considered statistically significant. All analyses were performed using Statistical Package for the Social Sciences version 26.0 (IBM Japan, Ltd., Tokyo, Japan).

Ethics and registration

This study was conducted from August 2024 to April 2025 in accordance with the Declaration of Helsinki. Ethical approval was obtained from the Institutional Review Board of Nagasaki University Hospital (approval number: 21091314), and research permission was obtained from all participating institutions. Written informed consent was obtained from all patients. The study was registered in the University Hospital Medical Information Network Clinical Trials Registry under the ID UMIN000054855 on July 8, 2024.

## Results

Patient characteristics

A total of 24 patients were enrolled by the end of the study period, at which point recruitment was closed. The patient characteristics are summarized in Table 1. The cohort included 10 men and 14 women, with a mean age of 42.2 years. The most common procedure was orthognathic surgery, performed in 17 cases. In addition, four patients underwent segmental mandibular resection for medication-related osteonecrosis of the jaw, and three patients underwent local resection for oral cancer.

**Table 1:** Patient characteristics MRONJ: medication-related osteonecrosis of the jaw, ABPC/SBT: ampicillin/sulbactam, CEZ: cefazolin, CLDM: clindamycin, SD: standard deviation

Factor	Category	Number of patients (mean ± SD)
Age (years)		42.2 ± 20.9
Sex	Man	10
	Woman	14
Primary disease	Jaw deformity	17
	MRONJ	4
	Oral cancer	3
Operation method	Orthognathic surgery	17
	Segmental mandibulectomy	4
	Cancer resection	3
Operation time (minutes)		211 ± 62.9
Antibiotic prophylaxis	ABPC/SBT (3-9 g/day, mean: 5.5 g/day)	12
	CEZ (2 g/day)	10
	CLDM (1.2 g/day)	2
Duration of postoperative antibiotic administration (days)		3.5 ± 2.2

Changes in salivary bacterial counts

**Figure 1:**
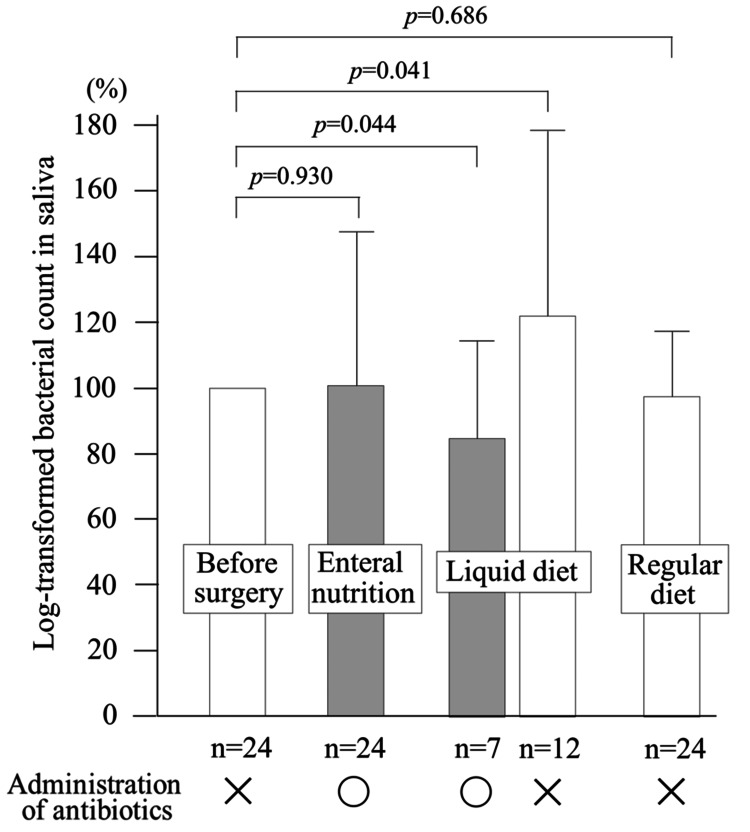
Changes in salivary bacterial counts according to feeding conditions During the enteral nutrition period, all patients received antibiotics, and there was no significant difference in bacterial counts compared to the preoperative period. In the liquid diet period, bacterial counts significantly decreased in patients receiving antibiotics, whereas they significantly increased in those who had completed antibiotic treatment. During the regular diet period, bacterial counts did not differ from the preoperative levels

Salivary bacterial counts at each dietary stage were expressed as a percentage, with the logarithmic value of the preoperative count set at 100. Setting the day of surgery as day 0, saliva samples were collected at an average of 2.1 days postoperatively during the enteral feeding phase, 4.2 days during the liquid diet phase, and 8.6 days during the solid diet phase. During the enteral feeding period, all patients received systemic antibiotic therapy, and the average bacterial count was 101. During the liquid diet period, patients who continued to receive antibiotics showed a significantly lower bacterial count (88), whereas those who had completed antibiotic therapy showed a significantly higher count (122). During the regular diet period, none of the patients were receiving antibiotics, and the average bacterial count was 98 (Figure 1).

These findings indicate that systemic antibiotic administration tended to suppress salivary bacterial counts. Furthermore, salivary bacterial counts increased during the liquid diet phase, particularly in the absence of antibiotics, but returned to preoperative levels during the regular diet phase.

## Discussion

This study demonstrated that systemically administered antibiotics effectively suppress the salivary bacterial count and that salivary bacterial levels are influenced by postoperative feeding status.

Oral and maxillofacial surgery is classified as clean-contaminated surgery, with surgical wounds continuously exposed to saliva containing bacteria. Therefore, meticulous wound closure to eliminate dead space and the use of prophylactic antibiotics are generally recommended [1,3,4]. Nevertheless, SSIs have been reported in various types of oral surgery, including minor procedures such as impacted third molar extraction, orthognathic surgery, and oral cancer resection [8-12].

Prolonged systemic antibiotic use carries risks, including adverse drug reactions and the emergence of antibiotic-resistant bacteria. To mitigate these risks, several studies have explored reducing salivary bacterial counts via local administration of antibiotics or disinfectants. Kirchner et al. [13] and Elledge et al. [14] reported that mouth rinsing with clindamycin significantly reduced salivary bacterial counts in healthy volunteers. Funahara et al. demonstrated that intraoral application of tetracycline ointment in patients undergoing oral cancer surgery with flap reconstruction reduced salivary bacterial counts for more than six hours postoperatively [15], and a subsequent randomized controlled trial showed that applying tetracycline ointment every six hours for 48 hours significantly reduced the incidence of SSI [16]. Imakiire et al. further reported that application of tetracycline ointment or povidone-iodine in intubated patients decreased bacterial counts not only in saliva but also in the fluid accumulating above the endotracheal tube cuff up to six hours after application [17,18]. However, even with local administration, prolonged use of antibiotics or disinfectants may contribute to the development of resistant bacteria.

In cases where surgical wounds are located intraorally, early postoperative mastication may compromise wound stability, potentially leading to dehiscence and bacterial contamination. Therefore, enteral feeding is often used until the wound stabilizes. However, the salivary bacterial count is influenced by the oral cavity’s self-cleaning mechanisms, including mastication and swallowing, and may increase in the absence of oral intake. Sakamoto et al. reported that in patients undergoing gastrointestinal surgery, salivary bacterial counts decreased during fasting but increased after the resumption of oral intake [19,20]. Similarly, Funahara et al. found a significant relationship between dietary intake and salivary bacterial levels in dependent elderly patients, with regular diet consumption contributing to reduced bacterial counts due to restored oral self-cleaning mechanisms [21]. Although enteral feeding and liquid diets may promote wound stability by minimizing mastication, they may also contribute to increased salivary bacterial load. To date, however, no studies have focused specifically on the relationship between postoperative feeding status and salivary bacterial count in oral and maxillofacial surgery.

In this study, salivary bacterial counts were measured during the preoperative period, enteral feeding phase, liquid diet phase, and regular diet phase. All patients received systemic antibiotics during the enteral feeding period. Although oral self-cleaning mechanisms were likely reduced during this time, salivary bacterial counts did not increase compared to preoperative levels, possibly due to a balance between the suppressive effect of systemic antibiotics and the absence of oral cleansing through mastication and swallowing. Honda et al. similarly reported that systemic antibiotics reduced salivary bacterial counts in pediatric patients undergoing cardiac surgery [22]. During the liquid diet phase, patients who were still receiving antibiotics showed decreased bacterial counts, while those who had completed antibiotic therapy exhibited a significant increase. During the regular diet phase, when no patients were receiving antibiotics, bacterial counts returned to preoperative levels.

These findings suggest that systemic antibiotics suppress salivary bacterial growth and that in the absence of adequate oral intake postoperatively, bacterial counts tend to increase. Therefore, if patients have not yet resumed a regular diet by the time systemic antibiotic administration is discontinued, additional measures, such as povidone-iodine gargling, may be necessary to reduce the increased salivary bacterial burden and help prevent SSI.

This study has several limitations. First, it was a preliminary study with a small sample size. The small sample size of 24 patients limits the generalizability of the findings in this study. As this was a preliminary, single-center study, the results should be interpreted with caution. In future research, it would be desirable to conduct larger multicenter studies based on formal power calculations to validate these findings and enhance the external validity of the conclusions. Additionally, data were not obtained from enterally fed patients who did not receive antibiotics, making it difficult to isolate the effect of enteral feeding alone on salivary bacterial counts. Nevertheless, this is the first study to examine the relationship between postoperative feeding status and salivary bacterial counts in oral and maxillofacial surgery. Future studies involving larger cohorts are warranted to explore this relationship in more detail and to establish effective oral care protocols for suppressing bacterial growth in saliva following surgery.

## Conclusions

This preliminary study demonstrated that systemic antibiotic administration effectively suppresses salivary bacterial counts in patients undergoing oral and maxillofacial surgery. In contrast, postoperative feeding status significantly influences bacterial levels, with an increase observed during the liquid diet phase, particularly after discontinuation of antibiotics. Salivary bacterial counts returned to preoperative levels upon resumption of a regular diet, likely due to restoration of the oral cavity's self-cleaning mechanisms. These findings suggest that additional oral hygiene interventions may be warranted during the interim period between antibiotic cessation and the start of regular oral intake to reduce the risk of SSI. While limited by a small sample size, this study provides important insights into the dynamic relationship between feeding status, antibiotic use, and salivary bacterial load, highlighting the need for further research to develop optimized perioperative oral care strategies.
